# Genetic characterization of *Rhipicephalus sanguineus* (*sensu lato*) ticks from dogs in Portugal

**DOI:** 10.1186/s13071-017-2072-1

**Published:** 2017-03-13

**Authors:** Filipe Dantas-Torres, Carla Maia, Maria Stefania Latrofa, Giada Annoscia, Luís Cardoso, Domenico Otranto

**Affiliations:** 1 Department of Immunology, Aggeu Magalhães Institute, Oswaldo Cruz Foundation (Fiocruz), Recife, Pernambuco 50740-465 Brazil; 2 Department of Veterinary Medicine, University of Bari, 70010 Valenzano Bari, Italy; 3 Current address: Global Health and Tropical Medicine, GHTM, Instituto de Higiene e Medicina Tropical, IHMT, Universidade Nova de Lisboa, UNL, Rua de Junqueira 100, 1349-008 Lisboa, Portugal; 4 Faculty of Veterinary Medicine, Universidade Lusófona de Humanidades e Tecnologias, Campo Grande, 376, 1749-024 Lisboa, Portugal; 5 Department of Veterinary Sciences, School of Agrarian and Veterinary Sciences, University of Trás-os-Montes e Alto Douro (UTAD), Vila Real, Portugal

**Keywords:** Brown dog ticks, *Rhipicephalus*, Dogs, Genetics, Morphology, Portugal

## Abstract

**Background:**

The taxonomic status of the brown dog tick *Rhipicephalus sanguineus* (*sensu stricto*) is a subject of on-going debate; there is a consensus that populations of this tick species should be referred to as *R. sanguineus* (*sensu lato*) until its taxonomic status is resolved. Recent genetic studies revealed the existence of more than one lineage of *R. sanguineus* (*s.l.*) in temperate countries. In this study, we assessed the genetic identity of ticks collected from rural dogs living in several areas located in all major geographical regions of Portugal.

**Methods:**

A total of 347 ticks were collected from rural dogs living in different regions of Portugal. These ticks were morphologically identified and partial mitochondrial 16S rRNA gene sequences (~300 bp) were obtained from representative specimens.

**Results:**

The ticks were morphologically identified as *Ixodes ricinus* (seven males and 27 females), *Rhipicephalus bursa* (one male), *Rhipicephalus pusillus* (one female) and *R. sanguineus* (*s.l.*) (two larvae, 101 nymphs, 108 males and 100 females). Partial mitochondrial 16S rRNA gene sequences were obtained from 58 *R. sanguineus* (*s.l.*) specimens, and all of them were genetically identified as belonging to the so-called temperate lineage of *R. sanguineus* (*s.l.*)

**Conclusions:**

These results strongly suggest that the temperate species of *R. sanguineus* (*s.l.*) is the only representative of this tick group found on dogs in Portugal. It also adds weight to the hypothesis that *Rhipicephalus turanicus* is not present in this country, although further investigations are necessary to confirm this.

**Electronic supplementary material:**

The online version of this article (doi:10.1186/s13071-017-2072-1) contains supplementary material, which is available to authorized users.

## Background

Ticks are important vectors of pathogens to companion animals, livestock and humans. Current global changes (e.g. climate changes, deforestation, changes in land use, urbanization, increased trade and travel) are affecting animal host populations worldwide [1], favouring the establishment of ticks and their associated pathogens into previously free areas.

The brown dog tick *Rhipicephalus sanguineus* (*sensu stricto*) is a species of major medical and veterinary significance [2]. The taxonomy of this tick species is a subject of an ongoing debate, mainly because there is no type-material and no *bona fide* morphological description [3, 4]. For this reason, there is a consensus that populations of this tick species should be referred to as *R. sanguineus* (*sensu lato*) (*s.l.*) until its taxonomic status is resolved.

Genetic studies have consistently reported the existence of two well-defined lineages within “*R. sanguineus*”: the southern lineage (also referred to as temperate species/lineage) and the northern lineage (tropical species/lineage) [5–11]. Available scientific data indicate that the situation regarding the taxonomic status of *R. sanguineus* (*s.l.*) in Europe is even more complicated. In particular, a higher diversity of cryptic species seems to occur in the Mediterranean region [8] as compared with Latin America [5–7]. Furthermore, the diversity of *Rhipicephalus* spp. appears to increase from West to East in the Eurasia [8]. Indeed, the so-called temperate species/lineage (also referred to as “*Rhipicephalus* sp. II” by Dantas-Torres et al. [8]) of *R. sanguineus* (*s.l.*) appears to be the only representative of this species group in western European countries such as Portugal and Spain [8]. On the other hand, additional operational taxonomic units (OTUs) (e.g. *Rhipicephalus* sp. I) and/or species (e.g. *Rhipicephalus turanicus*) are apparently present in countries such as Italy and Greece.

A considerable amount of genetic data have been generated from *R. sanguineus* (*s.l.*) ticks in recent years. However, only few studies have assessed the genetic variability of ticks from different regions within the same country (e.g. Brazil; [5]), in order to define which tick species/lineage is present. In this study, we assessed the genetic identity of *R. sanguineus* (*s.l.*) ticks collected from dogs from all major geographical regions of Portugal to assess two hypotheses: (i) *R. turanicus* is not found on dogs from Portugal; and (ii) the temperate species/lineage is the only representative of the *R. sanguineus* (*s.l.*) group infesting dogs in this country.

## Methods

From June 2013 to April 2014, ticks (*n* = 347) were collected from rural dogs living in different regions of Portugal (Table 1). The owners physically restrained dogs and ticks were collected manually and placed in labelled vials containing 70% ethanol. Identification was carried out under a stereomicroscope using morphological keys [12–14]. In particular, we considered all the morphological characters detailed in Dantas-Torres et al. [8], such as idiosoma, dorsal scutum, *basis capituli*, hypostomal dentition, female porose areas, female genital opening, spiracular plates, dorsal tail of spiracular plates, lateral and postmediam grooves, cervical pits, cervical fields, internal and external cervical grooves, marginal lines, male adanal and accessory plates, male caudal process, spur on trochanter I, and body colour. Ticks morphologically compatible with the description of Walker et al. [13] for “*R. sanguineus*” were referred to as “*R. sanguineus* (*s.l.*)”.

**Table 1:** Areas of Portugal surveyed in this study

Areas	Geographical coordinates
Alijó	41°17′0″N, 7°28′0″W
Azores	37°44′28″N, 25°40′32″W
Beja	38°1′0″N, 7°52′0″W
Bragança	41°48′20″N, 6°45′42″W
Faro	37°2′0″N, 7°55′0″W
Gondomar	41°9′0″N, 8°32′0″W
Gouveia	40°30′0″N, 7°36′0″W
Guarda	40°32′0″N, 7°20′0″W
Lourinhã	39°15′0″N, 9°19′0″W
Madeira	32°39′4″N, 16°54′35″W
Porto	41°9′0″N, 8°36′40″W
Sabugal	40°21′0″N, 7°5′0″W

A total of 61 ticks, including at least one tick from each geographical site, were selected for genetic analysis. DNA extraction was performed using a commercial kit (DNeasy Blood & Tissue Kit, Qiagen GmbH, Hilden, Germany), in accordance with the manufacturer’s instructions. Partial mitochondrial 16S rRNA (~300 bp) gene sequences were generated and analysed. Primers and PCR conditions have been described elsewhere [5]. Each reaction consisted of 4 μl of tick genomic DNA and 46 μl of PCR mix containing 2.5 mM MgCl_2_, 10 mM Tris-HCl (pH 8.3), and 50 mM KCl, 250 μM of each dNTP, 50 pmol of each primer and 1.25 U of AmpliTaq Gold (Applied Biosystems, California, USA). Approximately 100 ng of genomic DNA (with the exception of the no-template control) were added to each PCR. Amplified products were examined on 2% agarose gels stained with GelRed (VWR International PBI, Milan, Italy) and visualized on a GelLogic 100 gel documentation system (Kodak, New York, USA).

Amplified products were purified and sequenced, in both directions using the same primers as for PCR, employing the Big Dye Terminator v.3.1 chemistry in a 3130 genetic analyzer (Applied Biosystems, California, USA). The 16S rRNA gene sequences were aligned using ClustalW program [15] and compared with those available in GenBank using the BLASTn tool (http://blast.ncbi.nlm.nih.gov/Blast.cgi). The percentage of nucleotide variation (pairwise comparison – Pwc) amongst all haplotypes identified was calculated using the Kimura 2-parameter substitution model with gamma distributed rates among sites [16], implemented in the MEGA 6 software [17].

For phylogenetic analyses, we included sequences from each haplotype obtained herein as well as individual or consensus sequences (GenBank accession numbers: KC243835–KC243838, KC243843–KC243847, KC243851–KC243854, KC243855, KC243856–KC243867 and KC243871) for the other *Rhipicephalus* spp. (for more details, see Additional file 1: Table S1) available from a previous study [8]. In particular, consensus sequences from selected tick species were generated after alignment with ClustalW program [15] and using the BioEdit software [18]. A homologous gene sequence from *Ixodes ricinus* (GenBank JF928527) was used as the outgroup. Phylogenetic relationships were inferred by maximum likelihood analysis [16] with the general time reversible model in MEGA 6 [17]; bootstrap values are based on 8,000 replicates.

## Results

Morphologically, ticks were identified as *I. ricinus* (seven males and 27 females), *Rhipicephalus bursa* (one male), *Rhipicephalus pusillus* (one female), and *R. sanguineus* (*s.l.*) (two larvae, 101 nymphs, 108 males and 100 females). Most (all but 12) *R. sanguineus* (*s.l.*) ticks resembled (e.g. large, dark-coloured ticks, with males presenting elongated spiracular plates with narrow dorsal tails and females presenting typical U-shaped genital opening) the so-called temperate species (data not shown). Morphological variations in ticks collected from different regions and even within the same region were noticeable. In particular, some male ticks (ten from Bragança and two from Lourinhã) presented spiracular plates with short and large dorsal tails, which could resemble those of *R. turanicus*.

With regard to genetic data, a total of 59 partial 16S rRNA gene sequences were obtained; for two ticks no amplification was obtained. One of these sequences shared 98% identity with a sequence of *R. pusillus* available in GenBank (KC243855). The remaining 58 sequences were from ticks identified as *R. sanguineus* (*s.l.*) and were all genetically assigned to the temperate species/lineage, including specimens resembling morphologically *R. turanicus* (see Additional file 1: Table S1). BLAST analysis revealed that the eight haplotypes identified shared high nucleotide identity (99–100%) with those of *Rhipicephalus* sp. II (= temperate species/lineage) available in GenBank (KC243843–KC243846). Indeed, one haplotype was identical to haplotype II of *Rhipicephalus* sp. II previously identified in Portugal and in northern Italy (GenBank KC243844).

The new representative sequence types were named as haplotypes VI–XII. The haplotype VI was the most prevalent haplotype (*n* = 30; 51.7%) identified and found in all surveyed areas (Table 2), followed by haplotype II (*n* = 10; 17.2%). The nucleotide sequence variation, upon pairwise comparison, ranged from zero to 1.1% (mean 0.7%), and a high nucleotide difference (1.1%) was recorded between haplotypes II and X, which were obtained from specimens collected in the same area (Alijó). All representative new haplotypes obtained are available in the GenBank database under accession numbers: KY216135–KY216141.

**Table 2:** Ticks and haplotypes identified in areas of Portugal

Area	Nymph (*n*/haplotype)	Adult (*n*/haplotype)	
		Male	Female
Alijó	–	1/VI; 1/II; 1/VII	1/VI; 1/II; 1/VII; 2/X
Azores	1/IX	2/VI; 1/IX	–
Beja	1/VI	–	2/VI
Bragança	–	2/VI; 1/VII; 1/XI	1/VI; 1/VII
Faro	–	1/VI; 1/XII	1/VI
Gondomar	–	1/VI; 1/II; 1/VII	1/VIII
Gouveia	1/VI; 3/II	–	–
Guarda	2/VI	1/VI	1/VI
Lourinhã	–	1/VI; 1/VII	2/VI; 1/II
Madeira	1/VI	–	–
Porto	1/VIII	1/VI; 3/VIII	1/II; 1/VIII
Sabugal	–	2/VI; 1/II	5/VI; 1/II

**Fig. 1:**
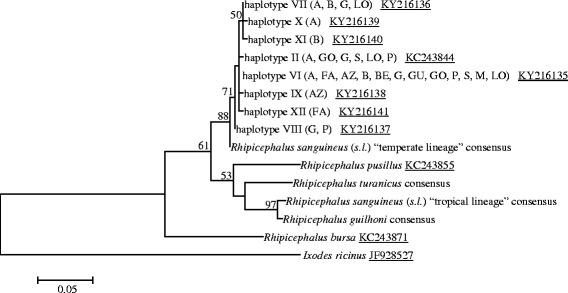
Phylogeny of *Rhipicephalus* spp. inferred from 16S rRNA sequences. Maximum-parsimony tree based on 16S rRNA sequences generated herein as well as either single haplotype or consensus sequences of *R. pusillus*, *R. bursa*, *R. sanguineus* (*s.l*.) “temperate lineage” (= *Rhipicephalus* sp. II; *Rhipicephalus* sp. morphotype 2), *R. turanicus*, *R. sanguineus* (*s.l.*) “tropical lineage”, and *R. guilhoni*. Geographical origins are reported in parentheses and GenBank accession numbers are underlined. Only bootstrap values ≥ 50% are indicated. *Abbreviations*: A, Alijó; AZ, Azores; BE, Beja; B, Bragança; FA, Faro; G, Gondomar; GO, Gouveia; GU, Guarda; LO, Lourinhã; M, Madeira; P, Porto; S, Sabugal

Phylogenetic analysis was concordant in clustering all haplotypes identified in the same clade with the consensus sequence of the temperate species/lineage, supported by a high bootstrap value, to the exclusion of other *Rhipicephalus* spp. (Fig. 1).

## Discussion

In this study, all *R. sanguineus* (*s.l.*) ticks collected from rural dogs living in different regions and areas of Portugal were morphologically compatible with the so-called temperate species/lineage. Twelve male ticks presented spiracular plates with large and short dorsal tails, resembling those of *R. turanicus* (data not shown), but all of them were genetically confirmed as indistinct from the temperate species/lineage. Our data further indicate that the “classic” morphological identification of *R. turanicus* based on only spiracular plates does not correlate with molecular findings. This also indicates that ticks previously identified as *R. turanicus* in Portugal (e.g. [19]) were actually *R. sanguineus* (*s.l.*) (temperate species/lineage), as already suggested by Santos-Silva et al. [20]. Indeed, our study indicates that the temperate species/lineage is the only representative of *R. sanguineus* (*s.l.*) infesting dogs in Portugal. The existence of a single species referred to as “*R. sanguineus*” with a high level of morphological polymorphism has been discussed elsewhere [20]. However, the existence of *R. turanicus* and even other species/lineages of *R. sanguineus* (*s.l.*) parasitizing other animal species in Portugal, particularly wildlife, cannot be ruled out and deserves further investigation.

Studies conducted in Latin America revealed the presence of two main lineages of *R. sanguineus* (*s.l.*) in the Neotropical region: tropical species (occurring from northern Mexico, Central America, and tropical areas of South America) and temperate species (found temperate and cold localities from the southern cone of South America) [5–7]. This notion is supported by more recent studies [8–10], indicating that ticks currently identified as “*R. sanguineus*” actually belong to at least two distinct species.

Because dogs are travelling with their owners (and with their ticks) around the world, the existence of two different lineages is intriguing. According to a recent study, the separation of these species seems to be driven by climate variables, particularly the annual mean temperature. Data indicate that the tropical species/lineage is present in areas with annual mean temperature > 20 °C, whereas the temperate species/lineage occurs in areas with annual mean temperature < 20 °C [11]. Whether ongoing climate changes will favour the establishment of populations of the tropical/lineage species in areas currently occupied exclusively by the temperate species/lineage and *vice versa* has yet to be determined.

The oldest record of a tick on a dog comes from ancient Egypt [21]. Ticks collected from a dog mummy found in a tomb surrounding a Roman fortress in El Dei were morphologically identified as belonging to the temperate species/lineage. Actually, the separation of the two main lineages of *R. sanguineus* (*s.l.*) might have occurred millions of years ago, as these species are reproductively isolated [22]. Moreover, differences in their complete mitochondrial genomes are estimated to be in the order of ~10% [23].

Finally, the known circulation of several pathogens (e.g. *Babesia vogeli*, *Cercopithifilaria* sp. II, *Ehrlichia canis*, *Hepatozoon canis*, *Rickettsia conorii* and *Rickettsia massiliae*) among *R. sanguineus* (*s.l.*) ticks, dogs, cats and foxes in Portugal [24–28] indicates that the temperate species/lineage is playing a role in the transmission of these agents in this country. Interestingly, a study conducted by Moraes-Filho et al. [29] suggested that the absence or scarcity of cases of canine monocytic ehrlichiosis due to *E. canis* in the southern cone of South America might be a result of vector incompetence of the *R. sanguineus* (*s.l.*) ticks that are found on dogs in this part of South America. Whether the different haplotypes of the temperate lineage of *R. sanguineus* (*s.l.*) found in Portugal and other European countries present different vector capacities for each of the abovementioned pathogens has yet to be determined.

## Conclusions

In conclusion, the data presented herein add weight to the hypotheses that the temperate species/lineage is the only representative of *R. sanguineus* (*s.l.*) in Portugal and that *R. turanicus* does not occur in this country.

## Additional file

Additional file 1:
**Table S1.** Sequences of *Rhipicephalus* spp. used in this study. (DOCX 16 kb)
