# Supplementary material for: Genetic characterization of Rhipicephalus sanguineus (sensu lato) ticks from dogs in Portugal
Source: Parasit Vectors. 2017 Mar 13;10:133. doi: 10.1186/s13071-017-2072-1 (PMC5346838; doi:10.1186/s13071-017-2072-1)
Supplement: Additional file 1: — Table S1. Sequences of Rhipicephalus spp. used in this study. (DOCX 16 kb) [file 13071_2017_2072_MOESM1_ESM.docx]

**Additional file 1. Table S1.** Sequences of *Rhipicephalus* spp. used in this study

| **Species** | **Haplotype** | **Accession numbers** | **Note** |
| --- | --- | --- | --- |
| *Rhipicephalus* *sanguineus* (*s.l.*) “temperate lineage” (=*Rhipicephalus* sp. II, *Rhipicephalus* sp. morphotype 2) | Haplotype 1 | KC243843 | Used to create consensus sequences |
|  | Haplotype 2* | KC243844 | Used to create consensus sequences |
|  | Haplotype 3 | KC243845 | Used to create consensus sequences |
|  | Haplotype 4 | KC243846 | Used to create consensus sequences |
|  | Haplotype 5 | KC243847 | Used to create consensus sequences |
|  | Haplotype 6* | KY216135 | New sequence |
|  | Haplotype 7* | KY216136 | New sequence |
|  | Haplotype 8 | KY216137 | New sequence |
|  | Haplotype 9 | KY216138 | New sequence |
|  | Haplotype 10 | KY216139 | New sequence |
|  | Haplotype 11 | KY216140 | New sequence |
|  | Haplotype 12 | KY216141 | New sequence |
| *Rhipicephalus turanicus* | Haplotype 1 | KC243856 | Used to create consensus sequences |
|  | Haplotype 2 | KC243857 | Used to create consensus sequences |
|  | Haplotype 3 | KC243858 | Used to create consensus sequences |
|  | Haplotype 4 | KC243859 | Used to create consensus sequences |
|  | Haplotype 5 | KC243860 | Used to create consensus sequences |
|  | Haplotype 6 | KC243861 | Used to create consensus sequences |
|  | Haplotype 7 | KC243862 | Used to create consensus sequences |
|  | Haplotype 8 | KC243863 | Used to create consensus sequences |
|  | Haplotype 9 | KC243864 | Used to create consensus sequences |
|  | Haplotype 10 | KC243865 | Used to create consensus sequences |
|  | Haplotype 11 | KC243866 | Used to create consensus sequences |
|  | Haplotype 12 | KC243867 | Used to create consensus sequences |
| *Rhipicephalus sanguineus* (*s.l.*) “tropical lineage” | Haplotype 1 | KC243835 | Used to create consensus sequences |
|  | Haplotype 2 | KC243836 | Used to create consensus sequences |
|  | Haplotype 3 | KC243837 | Used to create consensus sequences |
|  | Haplotype 4 | KC243838 | Used to create consensus sequences |
| *Rhipicephalus pusillus* | Haplotype 1 | KC243855 | Used in the phylogenetic analysis |
| *Rhipicephalus guilhoni* | Haplotype 1 | KC243851 | Used to create consensus sequences |
|  | Haplotype 2 | KC243852 | Used to create consensus sequences |
|  | Haplotype 3 | KC243853 | Used to create consensus sequences |
|  | Haplotype 4 | KC243854 | Used to create consensus sequences |
| *Rhipicephalus bursa* | Haplotype 1 | KC243871 | Used in the phylogenetic analysis |

* Haplotypes identified for specimens resembling morphologically *R. turanicus*: two belonged to haplotype 2; four to haplotype 6; and two to haplotype 7.
